# Mortality, Human Immunodeficiency Virus (HIV) Transmission, and Growth in Children Exposed to HIV in Rural Zimbabwe

**DOI:** 10.1093/cid/ciaa076

**Published:** 2020-01-24

**Authors:** Ceri Evans, Bernard Chasekwa, Robert Ntozini, Florence D Majo, Kuda Mutasa, Naume Tavengwa, Batsirai Mutasa, Mduduzi N N Mbuya, Laura E Smith, Rebecca J Stoltzfus, Lawrence H Moulton, Jean H Humphrey, Andrew J Prendergast, for the Sanitation Hygiene Infant Nutrition Efficacy (SHINE) Trial Team

**Affiliations:** 1 Zvitambo Institute for Maternal and Child Health Research, Harare, Zimbabwe; 2 Blizard Institute, Queen Mary University of London, London, United Kingdom; 3 Global Alliance for Improved Nutrition, Washington, District of Columbia, USA; 4 Department of Epidemiology and Environmental Health, School of Public Health and Health Professions, University at Buffalo, Buffalo, New York, USA; 5 Division of Nutritional Sciences, Cornell University, Ithaca, New York, USA; 6 Department of International Health, Johns Hopkins Bloomberg School of Public Health, Baltimore, Maryland, USA

**Keywords:** HIV transmission, mortality, growth, children HIV-exposed but uninfected, Africa

## Abstract

**Background:**

Clinical outcomes of children who are human immunodeficiency virus (HIV)–exposed in sub-Saharan Africa remain uncertain.

**Methods:**

The Sanitation Hygiene Infant Nutrition Efficacy (SHINE) trial evaluated improved infant and young child feeding (IYCF) and/or improved water, sanitation, and hygiene in 2 rural Zimbabwean districts with 15% antenatal HIV prevalence and > 80% prevention of mother-to-child transmission (PMTCT) coverage. Children born between February 2013 and December 2015 had longitudinal HIV testing and anthropometry. We compared mortality and growth between children who were HIV-exposed and HIV-unexposed through 18 months. Children receiving IYCF were excluded from growth analyses.

**Results:**

Fifty-one of 738 (7%) children who were HIV-exposed and 198 of 3989 (5%) children who were HIV-unexposed (CHU) died (hazard ratio, 1.41 [95% confidence interval {CI}, 1.02–1.93]). Twenty-five (3%) children who were HIV-exposed tested HIV positive, 596 (81%) were HIV-exposed uninfected (CHEU), and 117 (16%) had unknown HIV status by 18 months; overall transmission estimates were 4.3%–7.7%. Mean length-for-age z score at 18 months was 0.38 (95% CI, .24–.51) standard deviations lower among CHEU compared to CHU. Among 367 children exposed to HIV in non-IYCF arms, 147 (40%) were alive, HIV-free, and nonstunted at 18 months, compared to 1169 of 1956 (60%) CHU (absolute difference, 20% [95% CI, 15%–26%]).

**Conclusions:**

In rural Zimbabwe, mortality remains 40% higher among children exposed to HIV, vertical transmission exceeds elimination targets, and half of CHEU are stunted. We propose the composite outcome of “alive, HIV free, and thriving” as the long-term goal of PMTCT programs.

**Clinical Trials Registration:**

NCT01824940.

## Teaser

Children who are Human Immunodeficiency Virus (HIV)-exposed but uninfected (CHEU) in rural Zimbabwe had 40% higher mortality than HIV-unexposed children. HIV transmission rates exceeded elimination targets, and half of CHEU were stunted by 18 months of age, highlighting ongoing disparities in clinical outcomes.

**(See the Editorial Commentary by Slogrove and Powis on pages 595–7.)**

Before the availability of prevention of mother-to-child transmission (PMTCT) interventions in sub-Saharan Africa, human immunodeficiency virus (HIV) was a major contributor to child mortality. In a Zimbabwean birth cohort between 1997 and 2001, 80% of all child deaths were among HIV-exposed children [1]. Overall mother-to-child HIV transmission (MTCT) was 27%, but even children who were HIV exposed but uninfected (CHEU) had 3-fold higher mortality than children who were HIV unexposed (CHU) [1], and poor growth was common [2, 3]. Similar findings were reported from several sub-Saharan African settings [4].

An estimated 1.3 million children are born to women living with HIV annually [5]. The increasing availability of antiretroviral therapy (ART) in sub-Saharan Africa now means the vast majority of these children avoid HIV transmission [5, 6]; however, the outcomes of children exposed to HIV are poorly described. Since the global goal is to ensure that all children survive, thrive, and lead transformative lives [7], it is important to know how well PMTCT programs are reducing HIV transmission, and whether health and growth of CHEU have normalized. We therefore compared clinical outcomes between children who were HIV exposed and those who were HIV unexposed in rural Zimbabwe.

## METHODS

### Sanitation Hygiene Infant Nutrition Efficacy Trial

The Sanitation Hygiene Infant Nutrition Efficacy (SHINE) trial [8] recruited pregnant women from 2 rural Zimbabwean districts with 15% antenatal HIV prevalence, between November 2012 and March 2015. SHINE was a 2 × 2 factorial cluster randomized trial assessing the effects of improved infant and young child feeding (IYCF) and improved water, sanitation, and hygiene (WASH) on stunting and anemia at 18 months of age. Mother–infant pairs lived in clusters randomized to standard of care (SOC); IYCF (20 g small-quantity lipid-based nutrient supplement/day from 6 to 18 months of age, and complementary feeding counseling); WASH (pit latrine and 2 hand-washing stations, liquid soap and chlorine, a clean play space, and hygiene counseling); or IYCF + WASH (all interventions). Results were stratified by maternal HIV status and have been reported previously [9–12].

### Data Collection

Research nurses visited mothers in early pregnancy (~2 weeks after consent) and at 32 gestational weeks, to assess maternal and household characteristics. At baseline, mothers had height, weight, and mid-upper arm circumference (MUAC) measured, and household wealth assessed [13]. Infant birth date, weight, and delivery details were transcribed from health records. Home visits were scheduled at 1, 3, 6, 12, and 18 months postpartum. Infant weight, length, and MUAC were measured at every visit, and head circumference from 3 months of age. Nurses were standardized on anthropometric measurement every 6 months. Breastfeeding exclusivity was assessed at the 1-, 3-, and 6-month visits; continued breastfeeding was assessed at 12 and 18 months. Mortality was assessed through home visits, village health worker reports, and telephone calls, with date of death recorded where available. Maternal ART was documented at 3 timepoints (baseline, 32 gestational weeks, and 1 month postpartum) based on maternal report and review of handheld medical records.

### HIV Testing

Mothers were tested in pregnancy using a rapid test algorithm (Alere Determine HIV-1/2 test, and, if positive, INSTI HIV-1/2 test [BioLytical Laboratories]). Women testing positive had CD4 cell counts measured (Alere Pima Analyser) and were referred to local clinics. Viral loads were not measured. National PMTCT guidelines changed from World Health Organization (WHO) Option B (maternal ART from 14 gestational weeks until the end of breastfeeding) to Option B+ (lifelong ART for all pregnant and breastfeeding women) in November 2013. Women were encouraged to initiate co-trimoxazole and ART, to exclusively breastfeed, and to attend clinic at 6 weeks postpartum for early infant diagnosis and co-trimoxazole. The first-line ART regimen in Zimbabwe was 2 nucleoside reverse transcriptase inhibitors (NRTIs) and a nonnucleoside reverse transcriptase inhibitor; the second-line regimen was 2 NRTIs and a protease inhibitor. Women testing HIV negative in pregnancy were offered retesting at 18 months postpartum to detect seroconversion.

HIV-positive mothers were invited to enroll in a substudy in which children had blood collected at 1, 3, 6, 12, and 18 months for HIV testing; children of mothers not enrolling were only tested at 18 months. Children testing positive were referred to local clinics for ART. Children testing HIV negative at 18 months were classified as CHEU. Children not tested at 18 months due to caregiver refusal, defaulted visits, or loss to follow-up were classified as HIV unknown. Children who died were classified based on their HIV test result at the last trial visit before death, or as HIV unknown if they had never been tested or were not tested on the last trial visit before death. Before 18 months of age, HIV was diagnosed using DNA polymerase chain reaction (PCR) on dried blood spot samples or RNA PCR on plasma; and after 18 months, by PCR or rapid test algorithm, depending on samples provided. Inconclusive or discordant results were retested to confirm status; if no further samples were available or repeat testing was inconclusive, children were classified as HIV unknown.

### Statistical Analyses

We compared baseline characteristics between groups using multinomial and ordinal regression models with robust variance estimation, and Somers D for medians. We compared mortality between the HIV-exposed and HIV-unexposed groups using Cox proportional hazards models with robust variance estimation to account for clusters, adjusted for trial arm. When the exact date of infant death was unknown (11/249 [4%]), an estimated date was assigned using the conditional median among children who died beyond the timepoint when the child was last reported alive. To compare growth between CHEU and CHU, we used generalized estimating equations with an exchangeable working correlation structure, adjusted for trial arm. A log-binomial specification was used to estimate relative risks (RRs). For our primary growth analysis, we did not adjust for other baseline covariates, because our aim was to evaluate whether there were differences between CHEU and CHU regardless of whether these were driven by biological, socioeconomic, or demographic factors. As a secondary analysis, we adjusted for maternal and household baseline characteristics associated with the exposure (HIV exposure status) and outcomes (*z* scores) to determine if this had an effect on inferences. All analyses used Stata version 15.1 software (StataCorp, College Station, Texas).

There was no effect of IYCF or WASH on mortality [9, 10]; therefore, all children were included in mortality comparisons. Since IYCF improved growth and WASH had no effect [9, 10], children randomized to IYCF were excluded from growth analyses. Subgroup analyses were planned if there was a significant interaction between sex and HIV exposure. We undertook sensitivity analyses to estimate the proportion of HIV-unknown children likely to be infected, using a range of transmission scenarios.

### Study Oversight and Registration

Mothers provided written informed consent. The Medical Research Council of Zimbabwe and the Johns Hopkins Bloomberg School of Public Health approved the study protocol. The trial is registered at ClinicalTrials.gov (NCT01824940).

## RESULTS

Among 5280 pregnant women, there were 738 live births to 726 HIV-positive mothers and 3989 live births to 3937 HIV-negative mothers (Supplementary Figure 1). Baseline characteristics of mothers, households, and children are shown in Table 1. HIV-positive compared to HIV-negative mothers were on average 4 years older, spent fewer years at school, came from slightly smaller households, and were in lower household wealth quintiles; their infants had lower birthweight and institutional delivery rates. Among the 726 HIV-positive mothers, 402 (55%) had documented co-trimoxazole use during pregnancy and 587 (81%) had documented ART use; of these, approximately two-thirds were receiving tenofovir-based regimens. Only 2 mothers received protease inhibitors. Mean CD4 count was 474 (standard deviation [SD], 221) cells/μL; only 8% had CD4 counts < 200 cells/μL. Exclusive breastfeeding between birth and 6 months was high [14] and did not differ by HIV exposure (data not shown). However, total duration of breastfeeding was shorter among HIV-positive compared to HIV-negative mothers (mean, 14.8 [SD, 3.7] vs 16.4 [SD, 3.1] months).

**Table 1.** Maternal, Household, and Infant Baseline Characteristics

Baseline Characteristics^a^	HIV Exposed	HIV Unexposed	*P* Value
Mothers, No.	726	3937	
Infants, No.	738	3989	
Trial arm			.17
SOC	166/738 (22)	960/3989 (24)	
IYCF	158/738 (21)	963/3989 (24)	
WASH	205/738 (28)	996/3989 (25)	
IYCF+WASH	209/738 (28)	1070/3989 (27)	
Household characteristics			
Size, median (IQR)	4 (3–6)	5 (3–6)	<.001
Wealth quintile^b^			<.001
Lowest	191/712 (27)	680/3659 (19)	
Second	167/712 (23)	706/3659 (19)	
Third	136/712 (19)	743/3659 (20)	
Fourth	106/712 (15)	767/3659 (21)	
Highest	112/712 (16)	763/3659 (21)	
Maternal characteristics			
Age, y, mean (SD)	29.2 (6.3)	25.6 (6.6)	<.001
Height, cm, mean (SD)	160.2 (6.2)	160.1 (5.8)	.84
MUAC, cm, mean (SD)	26.2 (2.9)	26.4 (3.1)	.17
Completed schooling, y, mean (SD)	9.1 (2.1)	9.6 (1.8)	<.001
Parity, median (IQR)	2 (1–3)	2 (1–3)	<.001
Married	643/682 (94)	3546/3717 (95)	.27
Employed	67/710 (9)	311/3655 (9)	.38
Religion			.13
Apostolic	330/726 (45)	1762/3937 (45)	
Other Christian religion	288/726 (40)	1685/3937 (43)	
Other non-Christian religion	108/726 (15)	490/3937 (12)	
HIV disease severity and treatment			
CD4 count in pregnancy, cells/μL, mean (SD)^c^	474 (221)	NA	
CD4 count <200 cells/μL	46/613 (8)	NA	
Co-trimoxazole prophylaxis during pregnancy^d^	402/726 (55)	NA	
ART during pregnancy^e^	587/726 (81)	NA	
Tenofovir-based ART regimen	391/587 (67)	NA	
Zidovudine-based ART regimen	116/587 (20)	NA	
Other/unknown regimen^f^	80/587 (14)	NA	
Infant characteristics			
Female sex	367/733 (50)	1962/3974 (49)	.73
Birth weight, kg, mean (SD)	2.99 (0.50)	3.08 (0.50)	<.001
Birth weight <2500 g	84/651 (13)	326/3574 (9)	.004
Institutional delivery	544/649 (84)	3208/3604 (89)	.001
Vaginal delivery	610/659 (93)	3411/3664 (93)	.64
Breastfeeding duration, mo, mean (SD)	14.8 (3.7)	16.4 (3.1)	<.001

Data are presented as no./no. (%) unless otherwise indicated.

Abbreviations: ART, antiretroviral therapy; HIV, human immunodeficiency virus; IQR, interquartile range; IYCF, infant and young child feeding; MUAC, mid-upper arm circumference; NA, not applicable; SD, standard deviation; SOC, standard of care; WASH, water, sanitation, and hygiene.

^a^Baseline for mothers was 2 weeks after consent (~14 weeks’ gestation). Baseline for infants was at birth.

^b^Wealth index constructed as described in Chasekwa et al, PLoS One 2018; 13:e1099393.

^c^CD4 cell count at baseline visit, or at 32 gestational weeks’ visit if no baseline result.

^d^Any documented exposure to co-trimoxazole during pregnancy.

^e^Any documented exposure to ART during pregnancy.

^f^Includes non-tenofovir-based or non-zidovudine-based regimens, use of both tenofovir and zidovudine during pregnancy (including switching regimens), or undocumented ART regimen.

### Child Mortality

**Figure 1.**
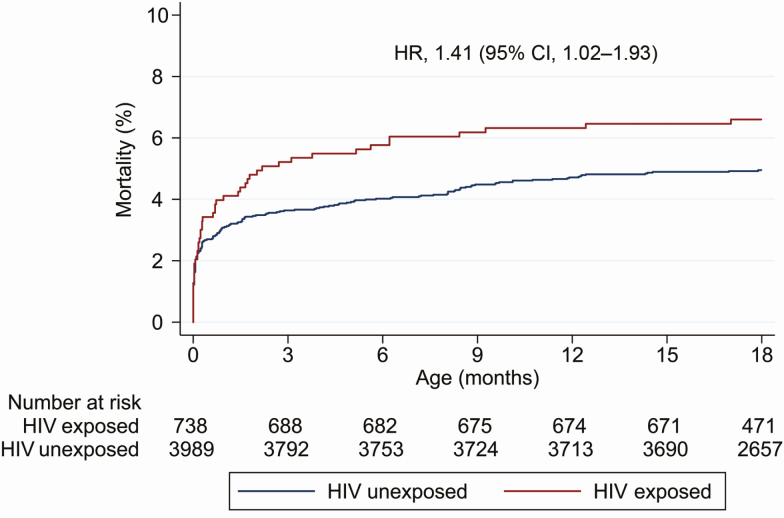
Cumulative hazard curves for mortality through 18 months. Kaplan-Meier curves are shown for children who were human immunodeficiency virus (HIV) exposed and children who were HIV unexposed. Hazard ratios were estimated by Cox regression analysis accounting for within-cluster correlation and adjusted for trial arm. Plot halted at 18 months. Abbreviations: CI, confidence interval; HIV, human immunodeficiency virus; HR, hazard ratio.

Among 738 children exposed to HIV, 51 (7% [95% confidence interval [CI], 5%–9%]) died compared to 198 of 3989 (5% [95% CI, 4%–6%]) children who were HIV unexposed (hazard ratio [HR], 1.41 [95% CI, 1.02–1.93]) (Figure 1). Of the 51 deaths in children exposed to HIV, 29 (57%) occurred before 28 days of age, 13 (25%) between 28 days and 6 months, 4 (8%) between 6 and 12 months, and 5 (10%) after 12 months. Of the 198 deaths in children who were HIV unexposed, 122 (62%) occurred before 28 days, 37 (19%) between 28 days and 6 months, 27 (14%) between 6 and 12 months, and 12 (6%) after 12 months. Child sex did not modify the effects of HIV exposure on mortality (*P* = .57).

Of the 51 deaths in children exposed to HIV, 46 (90%) occurred among children with unknown HIV status; 45 of 46 died without any HIV testing, and 1 child was HIV negative at the 1-month visit but died aged 6 months without further testing. Of the 5 children with known HIV status prior to death, 3 were HIV positive and 2 were HIV negative. Mortality was lower in children whose mothers received ART compared to those whose mothers did not receive ART in pregnancy (33/597 [6%] vs 18/141 [13%], respectively; HR, 0.41 [95% CI, .22–.76]). Overall mortality among ART-exposed children was similar to children who were HIV unexposed (HR, 1.11 [95% CI, .75–1.65]). By contrast, mortality among ART-unexposed children was almost 3-fold higher than among children who were HIV unexposed (HR, 2.74 [95% CI, 1.69–4.44]).

### Mother-to-Child Transmission of HIV

Among 738 children exposed to HIV, 25 (3%) were HIV positive, 596 (81%) were CHEU, and 117 (16%) had an unknown status at 18 months. MTCT was therefore 4% (25/621) among those with known HIV status. Baseline characteristics of CHEU and HIV-positive infants and their mothers are shown in Supplementary Table 1. HIV transmission by 18 months was 8 percentage points (95% CI, 2–15) lower in ART-exposed compared to ART-unexposed children (14/521 [3%] vs 11/100 [11%], respectively; RR, 0.26 [95% CI, .10–.63]). To bound our estimates of HIV transmission in the whole cohort, we undertook a sensitivity analysis, assuming various scenarios for HIV prevalence among the 117 children with unknown HIV status, which showed overall estimated HIV transmission of 4.3%–7.7% (Supplementary Table 2).

Among 3937 women testing HIV negative during pregnancy, 3387 (86%) were retested at 18 months postpartum; 41 of 3387 (1.2% [95% CI, .9–1.6]) had seroconverted during the postpartum period, meaning the 43 children born to these mothers became HIV exposed during breastfeeding. Of these 43 children, 9 (21%) tested HIV positive, 32 (74%) tested HIV negative, and 2 (5%) had an unknown HIV status at 18 months.

### Child Growth

Among 594 children exposed to HIV who were confirmed as CHEU at 18 months, 297 (50%) did not receive the IYCF intervention and were included in growth analyses. Among 3686 CHU assessed at 18 months, 1771 (48%) did not receive the IYCF intervention and were included in growth analyses (Table 2). The mean length-for-age *z* score (LAZ) was 0.38 (95% CI, .24–.51) SD lower in CHEU than in CHU, and stunting (LAZ < −2) was 16 percentage points (95% CI, 10–22) higher (150/297 [51%] vs 610/1771 [34%], respectively; RR, 1.46 [95% CI, 1.28–1.67]). Severe stunting (LAZ < −3) was 5 percentage points (95% CI, 1–10) higher in CHEU vs CHU (42/297 [14%] vs 155/1771 [9%], respectively; RR, 1.60 [95% CI, 1.15–2.21]). The mean weight-for-age *z* score (WAZ) was 0.17 (95% CI, .03–.32) SD lower in CHEU than in CHU, and underweight (WAZ < −2) was 6 percentage points (95% CI, 1–10) higher (48/297 [16%] vs 186/1765 [11%], respectively; RR, 1.51 [95% CI, 1.12–2.04]). By contrast, we found little evidence of differences in weight-for-length or wasting between groups at 18 months. The mean MUAC-for-age *z* score was 0.18 (95% CI, .07–.28) SD lower in CHEU compared to CHU at 18 months. The mean head circumference-for-age *z* score (HCZ) was 0.27 (95% CI, .14–.40) SD lower in CHEU compared to CHU, and microcephaly (HCZ < −2) was 4 percentage points (95% CI, 1–8) higher (29/296 [10%] vs 98/1757 [6%], respectively; RR, 1.74 [95% CI, 1.18–2.56]). Child sex did not modify the effects of HIV exposure on any growth outcomes (all *P* > .4). Inferences did not change after adjustment for baseline factors (Supplementary Table 3). Growth outcomes among HIV-positive children assessed at 18 months are shown in Supplementary Table 4.

**Table 2.** Growth Outcomes of Children Who Were Human Immunodeficiency Virus (HIV) Exposed but Uninfected Compared to Children Who Were HIV Unexposed at 18 Months of Age

Outcome	CHEU		CHU		Difference Between Means (95% CI)
	Mean (SD) *z* Score at 18-mo Visit				
	No.	*z* Score	No.	*z* Score	
LAZ	297	−1.97 (1.1)	1771	−1.58 (1.1)	−0.38 (−.51 to −.24)
WAZ	297	−0.93 (1.1)	1765	−0.75 (1.0)	−0.17 (−.32 to −.03)
WLZ	295	−0.03 (1.1)	1762	0.02 (1.0)	−0.04 (−.19 to .11)
MUAC-for-age *z* score	297	−0.17 (0.9)	1758	0.01 (0.9)	−0.18 (−.28 to −.07)
HCZ	296	−0.53 (1.1)	1757	−0.26 (1.1)	−0.27 (−.40 to −.14)
	Dichotomous Outcomes at 18-mo Visit				Relative Risk (95% CI)
	no./No.	%	no./No.	%	
Stunting (LAZ < −2)	150/297	51	610/1771	34	1.46 (1.28–1.67)
Severe stunting (LAZ < −3)	42/297	14	155/1771	9	1.60 (1.15–2.21)
Underweight (WAZ < −2)	48/297	16	186/1765	11	1.51 (1.12–2.04)
Wasting (WLZ < −2)	12/295	4	47/1762	3	1.52 (.78–2.97)
Microcephaly (HCZ < −2)	29/296	10	98/1757	6	1.74 (1.18–2.56)

Data were missing if not measured or implausible values.

Abbreviations: CHEU, children who are human immunodeficiency virus exposed but uninfected; CHU, children who are human immunodeficiency virus unexposed; CI, confidence interval; HCZ, head circumference-for-age *z* score; LAZ, length-for-age *z* score; MUAC, mid-upper arm circumference; SD, standard deviation; WAZ, weight-for-age *z* score; WLZ, weight-for length *z* score.

**Figure 2.**
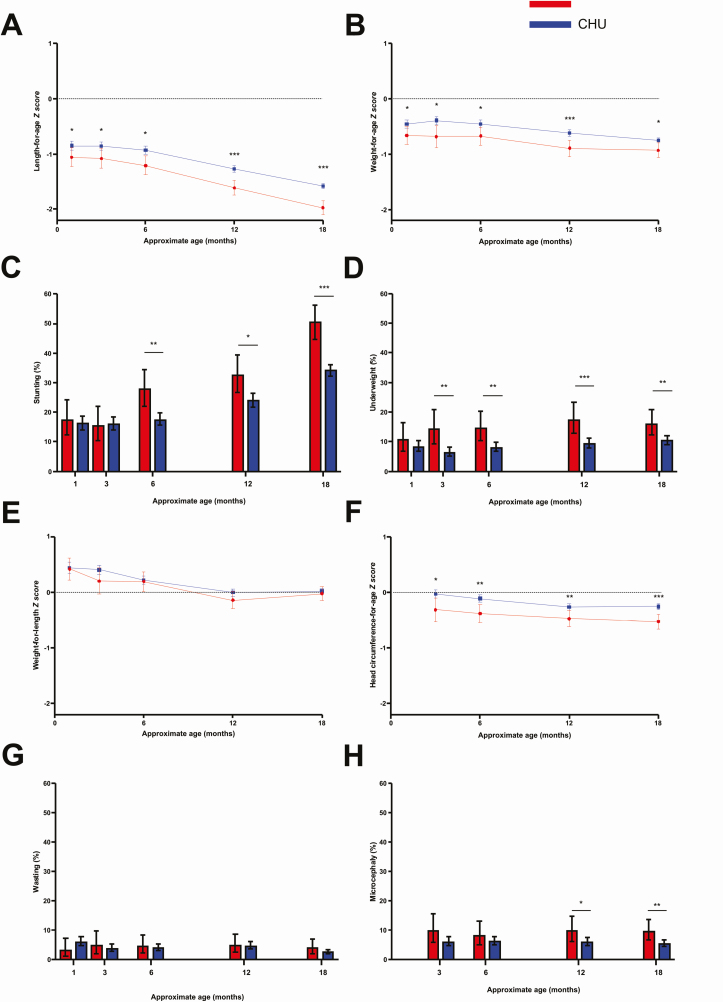
Growth outcomes of children who were human immunodeficiency virus (HIV) exposed but uninfected (CHEU) and children who were HIV unexposed (CHU) through 18 months of age. Length-for-age (*A*), weight-for-age (*B*), weight-for-length (*E*), and head circumference-for-age (*F*) *z* score growth trajectories in CHEU and CHU through 18 months. Data points are *z* score with 95% confidence intervals (CIs). Stunting (*C*), underweight (*D*), wasting (*G*), and microcephaly (*H*) in CHEU and CHU through 18 months. Data points are point prevalence percentages with 95% CIs. Number of children (CHEU/CHU) at each timepoint for length-for-age *z* score (*A*) and stunting (*C*): 176/1014 at 1 month, 116/1061 at 3 months, 215/1231 at 6 months, 220/1318 at 12 months, and 297/1771 at 18 months; weight-for-age *z* score (*B*) and underweight (*D*): 175/991 at Figure 2. Continued. 1 month, 160/1049 at 3 months, 217/1216 at 6 months, 221/1308 at 12 months, and 297/1765 at 18 months; weight-for-length (*E*) and wasting (*G*): 175/984 at 1 month, 159/1044 at 3 months, 215/1212 at 6 months, 220/1304 at 12 months, and 295/1762 at 18 months; head circumference-for-age *z* score (*F*) and microcephaly (*H*): 169/1038 at 3 months, 214/1232 at 6 months, 221/1321 at 12 months, and 296/1757 at 18 months (there was no head circumference measurement at 1 month of age). Stunting: length-for-age *z* score < −2; underweight: weight-for-age *z* score < −2; wasting: weight-for-length *z* score < −2; microcephaly: head circumference-for-age *z* score < −2. Only children in non–infant and young child feeding trial arms were included. *P < .05, **P < .01, ***P < .001. Full details including CIs appear in Supplementary Table 5.

To understand the timing and pattern of growth impairment, we assessed longitudinal anthropometry in children with available data at 1, 3, 6, and 12 months of age (Figure 2 and Supplementary Table 5). Growth failure began early: Differences in length-for-age and weight-for-age between CHEU and CHU were already apparent by 1 month of age. Weight-for-length in both groups was similar to WHO standards between 1 and 18 months, and wasting was uncommon. Differences in head circumference between groups were apparent at every timepoint.

### Alive, HIV Free, and Nonstunted

**Figure 3.**
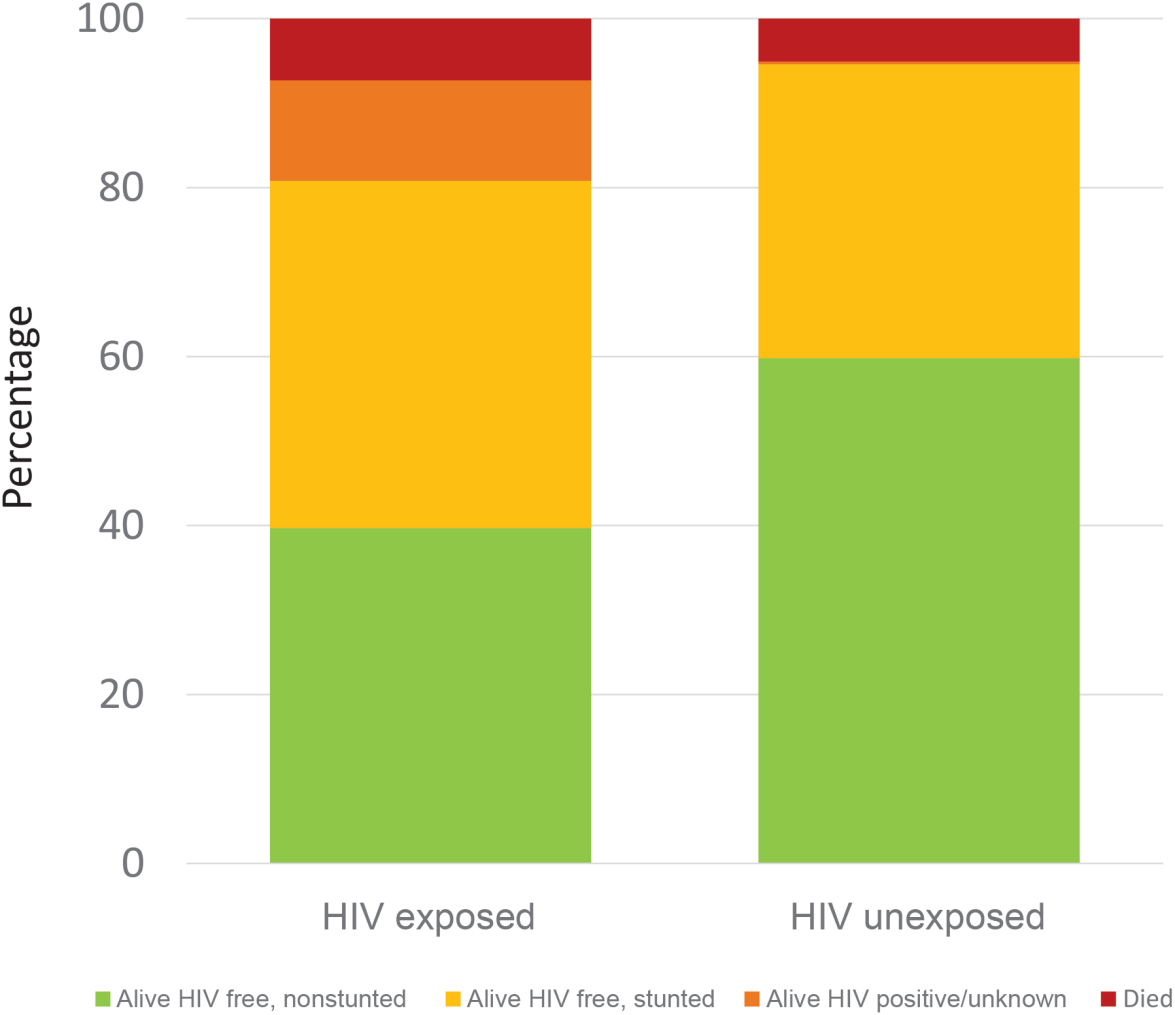
Proportion of children who were alive, human immunodeficiency virus (HIV) free, and nonstunted at 18 months. Only children in non–infant and young child feeding trial arms were included. HIV exposed, n = 371; HIV unexposed, n = 1956. Among 1956 children who were HIV unexposed, 21 children were born to mothers who tested HIV negative in pregnancy but HIV positive at 18 months postpartum, meaning they became HIV exposed during breastfeeding; of these, 5 of 1956 (0.26%) were HIV positive at 18 months and 1 of 1956 (0.05%) was HIV unknown. Among the 1927 HIV-negative mothers of the 1956 children who were HIV unexposed, 266 (14%) did not undergo repeat HIV testing at 18 months; it is therefore possible that additional children in the HIV-unexposed group became postnatally exposed and therefore infected. The HIV incidence among groups tested was 1.2%, and HIV transmission occurred in 21% of children born to postpartum seroconverting mothers. Assuming similar circumstances, approximately 3 of the 266 mothers may have been HIV positive at 18 months, and there would be < 1 expected vertical transmission.

To provide a composite measure of whether children exposed to HIV survived and thrived, we determined the proportion in non-IYCF arms who were known to be alive, HIV free, and nonstunted at the 18-month trial endpoint. Among 371 HIV-exposed live births, 147 (40% [95% CI, 35%–45%]) were known to be alive, HIV free, and nonstunted, compared to 1169 of 1956 (60% [95% CI, 58%–62%]) HIV-unexposed live births (absolute difference, 20% [95% CI, 15%–26%; RR, 0.66 [95% CI, .58–.76]) (Figure 3).

## DISCUSSION

Although increased PMTCT coverage in sub-Saharan Africa has substantially reduced the number of children living with HIV, mortality and growth outcomes among children exposed to HIV remain uncertain. Our study in rural Zimbabwe has 3 key findings. First, despite > 80% PMTCT coverage, mortality in children exposed to HIV remains 40% higher than in children not exposed to HIV, and occurs soon after birth; second, the proportion of children with HIV-positive or HIV-unknown status at 18 months remained above pediatric HIV elimination targets; and, third, growth of CHEU was significantly poorer than CHU, with more than half becoming stunted by 18 months. These findings from a rural setting with high antenatal HIV prevalence, similar ART coverage to global estimates [5], and high exclusive [14] and prolonged breastfeeding rates highlight the urgent action that is needed to ensure that children born to HIV-positive mothers survive and thrive.

Overall, 5% of children died by 18 months. Mortality was predominantly early, with half of all deaths occurring in the first 9 days after birth. We found 40% increased mortality among children born to HIV-positive compared to HIV-negative mothers. Strikingly, 90% of HIV-exposed children who died had no HIV testing, because deaths mostly occurred before early infant diagnosis at 4–6 weeks of age. We were therefore unable to ascertain whether the excess mortality was due to HIV transmission, or whether CHEU have a higher risk of death. The 3-fold higher mortality among children exposed to HIV whose mothers did not receive ART was similar to findings in pre-ART Zimbabwe [1]. By contrast, children exposed to HIV whose mothers were receiving ART had similar mortality to HIV-unexposed children. Maternal ART, as well as reducing HIV transmission, may also benefit CHEU through decreased exposure to maternal viremia, immunosuppression, and inflammation [15–17]. Regardless of whether antenatal ART reduces mortality directly by reducing HIV transmission or indirectly through other mechanisms, these findings reinforce the importance of ensuring that all HIV-positive pregnant women receive ART.

Among children with known HIV status at 18 months, MTCT was 4%. Despite PMTCT availability, one-fifth of HIV-positive women had no evidence of ART use during pregnancy; among these women, MTCT was 11%. A worrying finding is that 16% of children had an unknown HIV status, even in the context of a clinical trial where longitudinal HIV testing was offered; this proportion is likely even higher in programmatic settings [5]. We therefore believe the 4% transmission rate is an underestimate, since untested children may have a higher transmission risk. Our sensitivity analysis estimated MTCT to be 4.3%–7.7% by 18 months; we suspect the true transmission rate lies toward the upper end of these estimates, highlighting the considerable work that is still needed to prevent fallout from the PMTCT cascade. Another alarming finding is ongoing high HIV incidence among breastfeeding women, and the high MTCT rate (21%) among these mother–infant pairs, which is similar to estimates in Zimbabwe between 1997 and 2001 [18]. Our collective findings argue strongly for additional interventions to improve infant outcomes. Antenatal and postnatal ART adherence support, more effective virological suppression during pregnancy and breastfeeding, HIV testing before 4–6 weeks of age, and enhanced infant postexposure prophylaxis may all be beneficial.

Despite avoiding HIV infection, half of all CHEU were stunted at 18 months of age. Stunting is associated with increased child mortality, reduced academic outcomes and lifelong earnings, and intergenerational effects on health and human capital [19–22]. In areas of high HIV prevalence, this burden of stunting among CHEU therefore has enormous population impact. CHEU had 60% relative increase in severe stunting (LAZ < −3), which is concerning because the association between LAZ and mortality rises exponentially as LAZ falls [22]. Overall, growth impairment occurred early, with little recovery thereafter. CHEU had lower birthweight than CHU, and length was significantly lower when first measured at 1 month of age, consistent with an intrauterine effect of HIV exposure on ponderal and linear growth. Our linear growth findings are consistent with several other large cohorts in sub-Saharan Africa [23–26]. CHEU also had reduced weight-for-age, MUAC, and head circumference, highlighting the multiple anthropometric deficits that occur in this vulnerable group. Differences in head circumference may be particularly important given our recent finding of poorer neurodevelopment at 2 years of age in CHEU from this same cohort [27].

The global ambition that all children should survive, thrive, and lead transformative lives [7] is therefore particularly challenging in areas of high antenatal HIV prevalence. To focus efforts on this goal, we propose that PMTCT programs should strive for the composite outcome of “alive, HIV free, and thriving” as an indicator of success, rather than HIV-free survival alone. Only 40% of children exposed to HIV were known to be alive, HIV-free, and nonstunted at 18 months, compared to 60% of CHU. This provides clear evidence that most CHEU are not reaching their full potential. Our recent finding, from this same cohort, that CHEU were particularly responsive to improved feeding [10], provides optimism that children are amenable to interventions, despite their vulnerability [28].

This study has strengths and limitations. This is one of the largest comparisons of CHEU and CHU, with both groups drawn from the same rural communities. We restricted growth analyses to children not receiving the trial IYCF intervention. The population was well characterized, had high rates of follow-up at 18 months, and underwent longitudinal HIV testing, thereby reducing bias from attrition and HIV misclassification. However, we were unable to determine HIV status among a substantial proportion of children, including most of those who died, meaning the overall HIV transmission rate was uncertain. The trial did not provide HIV care or dispense ART, but we encouraged attendance at local clinics, where PMTCT coverage was high but not universal. We promoted exclusive breastfeeding to high levels [14], which may have reduced HIV transmission and improved health outcomes.

In summary, our study confirms a substantial ongoing impact of maternal HIV on child health in sub-Saharan Africa. Despite increasing availability of ART, mortality remains 40% higher among children exposed to HIV, MTCT rates are above elimination targets, and half of CHEU are stunted. We propose the composite outcome of “alive, HIV free, and thriving” as the long-term goal, with indicators such as stunting prevalence used to monitor PMTCT programs. Our findings highlight the ongoing disparity between CHEU and CHU, and the need for additional interventions to ensure that all children survive and thrive.

## Supplementary Data

Supplementary materials are available at *Clinical Infectious Diseases* online. Consisting of data provided by the authors to benefit the reader, the posted materials are not copyedited and are the sole responsibility of the authors, so questions or comments should be addressed to the corresponding author.

ciaa076_suppl_Supplementary_Appendix

## Notes

***Author contributions.*** C. E. led data analysis and interpretation of this substudy and wrote the first draft of the manuscript. B. C., M. N. N. M., L. E. S., L. H. M., J. H. H., and A. J. P. contributed to data analysis and interpretation of this substudy. R. N., M. N. N. M., R. J. S., L. H. M., J. H. H., and A. J. P. coauthored the original protocols and contributed to design and implementation of the trial and data analysis and interpretation. R. N. developed and managed all information technology, data management, and data analysis. F. D. M. and B. M. supervised all data collection. K. M. managed the laboratory and led all human immunodeficiency virus testing and interpretation. N. V. T. managed field operations. L. H. M. was the senior statistician. J. H. H. was the principal investigator of the Sanitation Hygiene Infant Nutrition Efficacy (SHINE) trial. A. J. P. directed all clinical and laboratory aspects of the trial and managed the data collection and laboratory teams. All authors contributed to, reviewed, and approved this manuscript.

***Acknowledgments.*** The authors heartily thank all the mothers, babies, and their families who participated in the SHINE trial. The authors gratefully acknowledge the leadership and staff of the Ministry of Health and Child Care in Chirumanzu and Shurugwi districts and Midlands Province (especially environmental health, nursing, and nutrition) for their roles in operationalization of the study procedures. The authors acknowledge the Ministry of Local Government officials in each district who supported and facilitated field operations. The authors are particularly indebted to Phillipa Rambanepasi and her team for proficiently managing all the finances and Virginia Sauramba for managing compliance issues. Finally, the authors are very thankful for our program officers at the Bill & Melinda Gates Foundation and UK Aid who enthusiastically worked with them over a long period of time to make SHINE happen.

***Financial support*.** This work was supported by the Bill & Melinda Gates Foundation (grant numbers OPP1021542 and OPP1143707); the United Kingdom Department for International Development (DFID/UK Aid); the Wellcome Trust (grant numbers 093768/Z/10/Z to A. J. P., 108065/Z/15/Z to A. J. P., and 210807/Z/18/Z to C. E.); Swiss Agency for Development and Cooperation; US National Institutes of Health (grant number 2R01HD060338-06); and the United Nations Children’s Fund (grant number PCA-2017-0002).

***Potential conflicts of interest.*** The authors: No reported conflicts of interest. All authors have submitted the ICMJE Form for Disclosure of Potential Conflicts of Interest.
