# Supplementary material for: Mortality, Human Immunodeficiency Virus (HIV) Transmission, and Growth in Children Exposed to HIV in Rural Zimbabwe
Source: Clin Infect Dis. 2020 Jan 24;72(4):586–94. doi: 10.1093/cid/ciaa076 (PMC7884806; doi:10.1093/cid/ciaa076)
Supplement: ciaa076_suppl_Supplementary_Appendix [file ciaa076_suppl_supplementary_appendix.docx]

**SUPPLEMENTAY APPENDIX: SUPPLEMENTARY FIGURES AND TABLES**

**Mortality, HIV transmission and growth in children exposed to HIV in rural Zimbabwe**

Ceri Evans, Bernard Chasekwa, Robert Ntozini, Florence D. Majo, Kuda Mutasa, Naume Tavengwa, Batsirai Mutasa, Mduduzi N. N. Mbuya, Laura Smith, Rebecca J. Stoltzfus, Lawrence H. Moulton, Jean H. Humphrey, Andrew J. Prendergast, for the SHINE Trial Team

**1** woman enrolled once for two pregnancies in error

**11** women enrolled twice in error

**5280** pregnant women enrolled from 210 randomized clusters^1^

**5270** pregnant women enrolled from 211 randomized clusters^1^

**49** women exited

**90** lost to follow up antenatally

**82** additional fetuses

**252** miscarriages

**113** stillbirths

**4** maternal deaths

**116** fetuses of **114** HIV-unknown mothers

**3989** live infants born to **3937** HIV-negative mothers included in **mortality analyses**

**738** live infants born to **726** HIV-positive mothers included in **mortality and HIV transmission analyses**

**51** infant deaths

**18** lost to follow up

**1** exit

**18 months**

**668** children HIV-unexposed assessed for trial primary outcome

**18 months**

**3686** children HIV-unexposed assessed for trial primary outcome

**198** infant deaths

**100** lost to follow up

**5** exits

**18 months**

**297** children HIV-exposed uninfected included in **growth analyses**

**297** in IYCF arms

**18 months**

**1771** children HIV-unexposed included in **growth analyses**

**1915** in IYCF arms

**22** HIV-positive

**52** HIV-unknown

**18 months**

**594** children HIV-exposed uninfected assessed for trial primary outcome

**Supplementary Figure 1. Flow of participants through the trial by maternal HIV status.**

^1^212 clusters were randomly assigned, 53 in each of the four trial groups. After randomisation, one cluster was excluded because it was in an urban area, one was excluded because the village health workers covering it mainly had clients outside the study area, and two more were merged on the basis of subsequent data for village health worker coverage. Three new cluster designations were created because of anomalies in the original mapping. For two of these clusters, the trial group was clear; the third contained areas that were in two trial groups, and was assigned to the under-represented group, resulting in 53 clusters in each group. All these changes occurred before enrolment began. When enrolment was completed, however, no women were enrolled in one cluster in the SOC group and thus 211 clusters were available for analysis.

| **Baseline characteristics^1^** | **HIV-positive** | **HIV-exposed uninfected** |
| --- | --- | --- |
| Mothers [N] | 25 | 587 |
| Infants [N] | 25 | 596 |
| **Trial arm^2^**  SOC  IYCF  WASH  IYCF+WASH  **Household characteristics** | 3/25 (12%)  3/25 (12%)  10/25 (40%)  9/25 (36%) | 137/587 (23%)  135/587 (23%)  159/587 (27%)  156/587 (27%) |
| Size; median (IQR) | 4 (3, 6) | 4 (3, 6) |
| Wealth quintile:^3^ |  |  |
| Lowest | 8/25 (32%) | 153/582 (26%) |
| Second | 4/25(16%) | 134/582 (23%) |
| Middle | 4/25 (16%) | 116/582 (20%) |
| Fourth | 6/25 (24%) | 84/582 (14%) |
| Highest | 3/25 (12%) | 95/582 (16%) |
| **Maternal characteristics** |  |  |
| Age, years; mean (SD) | 26 .8 (3.8) | 29.5 (6.3) |
| Height, cm; mean (SD) | 159.9 (6.9) | 160.1 (6.1) |
| MUAC, cm; mean (SD) | 24.5 (2.6) | 26.4 (2.9) |
| Completed schooling, years; mean (SD) | 9.5 (1.6) | 9.2 (2.1) |
| Parity; median (IQR) | 2 (1, 2.5) | 2 (1, 3) |
| Married | 22/23 (96%) | 516/550 (94%) |
| Employed | 2/25 (8%) | 53/574 (9%) |
| Religion: |  |  |
| Apostolic | 8/25 (32%) | 268/587 (46%) |
| Other Christian religions | 12/25 (48%) | 232/587 (40%) |
| Other non-Christian religions | 5/25 (20%) | 87/587 (15%) |
| HIV disease severity and treatment: |  |  |
| CD4 count in pregnancy, cells/uL; mean (SD)^4^ | 398 (200) | 482 (219) |
| CD4 count <200 cells/uL | 4/21 (19%) | 32/491 (7%) |
| Co-trimoxazole prophylaxis during pregnancy^5^ | 8/25 (32%) | 355/587 (60%) |
| Antiretroviral therapy during pregnancy^6^ | 14/25 (56%) | 499/587 (85%) |
| Tenofovir-based ART regimen | 6/14 (43%) | 341/587 (58%) |
| Zidovudine-based ART regimen | 5/14 (36%) | 93/587 (16%) |
| Other/unknown regimen^7^ | 3/14 (21%) | 153/587 (26%) |
| **Infant characteristics** |  |  |
| Female | 13/25 (52%) | 296/596 (50%) |
| Birth weight, kg; mean (SD) | 2.95 (0.50) | 3.01 (0.47) |
| Birth weight <2500 g | 5/21 (24%) | 62/552 (11%) |
| Institutional delivery | 21/25 (84%) | 469/553 (85%) |
| Vaginal delivery | 24/25 (96%) | 518/562 (92%) |

**Supplementary Table 1. Maternal, household, and infant baseline characteristics of HIV-exposed families**

^1^ Baseline for mothers was 2 weeks after consent (~14 weeks gestation). Baseline for infants was at birth.

^2^ SOC = standard of care; IYCF = infant and young child feeding; WASH = water and sanitation/hygiene.

^3^ Wealth index constructed as described in Chasekwa B, Maluccio JA, Ntozini R, *et al.* Measuring wealth in rural communities: lessons from the Sanitation, Hygiene, Infant Nutrition Efficacy (SHINE) trial. PLoS One 2018; 13: e0199393.

^4^ CD4 count at baseline visit, or at 32 gestational week visit if no baseline result.

^5^Any documented exposure to co-trimoxazole during pregnancy.

^6^ Any documented exposure to antiretroviral therapy during pregnancy.

^7^ Includes non-tenofovir- or zidovudine-based regimens, use of both tenofovir and zidovudine during pregnancy (including switching regimens), or undocumented antiretroviral therapy regimen.

SD: standard deviation; IQR: interquartile range; MUAC: mid-upper arm circumference; ART: antiretroviral therapy.

| **Assumption** | **Assumed transmission rate amongst HIV-unknown group** | **HIV-unknown infants assumed to be HIV-positive** | **Known HIV transmissions** | **n/N** | **Overall transmission rate** | **Comments** |
| --- | --- | --- | --- | --- | --- | --- |
| Only include those with a known HIV status | N/A | N/A | 25 | 25/621 | 4.0% |  |
| HIV-unknown infants have same transmission rate as the known group (4.0%) | 4% | 5/117 | 25 | 30/738 | 4.1% | Likely to underestimate actual transmission because HIV-unknown children likely to have a higher transmission rate (note that causes of being HIV-unknown include infant death and disengagement of mothers in HIV testing – both may be associated with HIV transmission) |
| HIV-unknown infants exposed to maternal ART (N=76) have a transmission rate of 2.7%; HIV-unknown infants not exposed to maternal ART (N=41) have a transmission rate of 11.0%, based on SHINE transmission data | 6% | 7/117 | 25 | 32/738 | 4.3% |  |
| Assume HIV-transmission amongst HIV-unknown is 2x higher than amongst those with known statuses | 8% | 9/117 | 25 | 34/738 | 4.6% |  |
| Assume HIV-transmission amongst HIV-unknown is 3x higher than amongst those with known statuses | 12% | 14/117 | 25 | 39/738 | 5.3% |  |
| Assume HIV-transmission amongst HIV-unknown is 5x higher than amongst those with known statuses | 20% | 23/117 | 25 | 48/738 | 6.5% |  |
| HIV-unknown infants have a transmission rate of 27% (assuming all mothers of HIV-unknown infants defaulted treatment; based on 24mo transmission rate in a breastfeeding population in pre-ART Zimbabwe, ZVITAMBO^1^) | 27% | 32/117 | 25 | 57/738 | 7.7% | Likely to overestimate actual transmission because 65% of HIV-unknown children had documented antenatal ART exposure |

1.Marinda E, Humphrey JH, Iliff PJ, et al. Child mortality according to maternal and infant HIV status in Zimbabwe. *Pediatr Infect Dis J.* 2007;26(6):519-526.

**Supplementary Table 2. Sensitivity analysis to estimate overall HIV prevalence among HIV-exposed children**

|  | **HIV-exposed uninfected** | | | **HIV-unexposed** | |  |  |
| --- | --- | --- | --- | --- | --- | --- | --- |
|  | **Mean (SD) Z-score at 18-month visit** | | | | | **Difference between means (95%CI)** | **Adjusted difference between means (95%CI)*** |
|  | **N** | **Z-score** | **N** | | **Z-score** |  |  |
| **Length-for-age Z-score** | 297 | -1.97 (1.1) | | 1771 | -1.58 (1.1) | -0.38 (-0.51, -0.24) | -0.39 (-0.51, -0.27) |
| **Weight-for-age Z-score** | 297 | -0.93 (1.1) | | 1765 | -0.75 (1.0) | -0.17 (-0.32, -0.03) | -0.17 (-0.31, -0.03) |
| **Weight-for-length Z-score** | 295 | -0.03 (1.1) | | 1762 | 0.02 (1.0) | -0.04 (-0.19, 0.11) | 0.00 (-0.14, 0.14) |
| **Mid-upper arm circumference-**  **for-age Z-score** | 297 | -0.17 (0.9) | | 1758 | 0.01 (0.9) | -0.18 (-0.28, -0.07) | -0.15 (-0.26 -0.04) |
| **Head circumference-for-age**  **Z-score** | 296 | -0.53 (1.1) | | 1757 | -0.26 (1.1) | -0.27 (-0.40, -0.14) | -0.31 (-0.44, -0.18) |
|  | **Dichotomous outcomes at 18-month visit** | | | | | **Relative risk (95%CI)** | **Adjusted relative risk (95%CI)*** |
|  | **n/N** | **Percentage** | **n/N** | | **Percentage** |  |  |
| **Stunting** | 150/297 | 51% | | 610/1771 | 34% | 1.46 (1.28, 1.67) | 1.49 (1.31, 1.70) |
| **Severe stunting** | 42/297 | 14% | | 155/1771 | 9% | 1.60 (1.15, 2.21) | 1.71 (1.29, 2.27) |
| **Underweight** | 48/297 | 16% | | 186/1765 | 11% | 1.51 (1.12, 2.04) | 1.58 (1.21, 2.08) |
| **Wasting** | 12/295 | 4% | | 47/1762 | 3% | 1.52 (0.78, 2.97) | 1.30 (0.66, 2.58) |
| **Microcephaly** | 29/296 | 10% | | 98/1757 | 6% | 1.74 (1.18, 2.56) | 1.80 (1.24, 2.61) |

**Supplementary Table 4. Adjusted differences between growth outcomes of children who are HIV-exposed uninfected (CHEU) compared to children who are HIV-unexposed uninfected (CHU) at 18 months of age.**

Stunting: length-for-age Z-score < -2; severe stunting: length-for-age Z-score < -3; underweight: weight-for-age Z-score < -2; wasting: weight-for-length Z-score < -2; microcephaly: head circumference-for-age Z-score < -2; 95%CI: 95% confidence interval. Data were missing if not measured or implausible values. All models were adjusted for the following covariates: child sex, child age at 18 month visit, calendar month of birth, and trial arm. The following covariates were offered into models, and were retained in the final adjusted model if they were associated with the exposure (HIV exposure status) and the continuous outcome (Z-score) on univariable analysis: maternal age, height, parity, religion, mid-upper arm circumference (MUAC), education marital status and employment status, and household wealth and size. The length-for-age and stunting models also including a variable for outlying study nurses based on length-for-age Z-score assessment. The variables retained for each outcome were as follows. Length-for-age/stunting: sex, age at 18 month visit, calendar month of birth, trial arm, maternal height, maternal religion, household wealth, outlying study nurse. Severe stunting: sex, age at 18 month visit, calendar month of birth, trial arm, maternal height, maternal religion, household wealth. Weight-for-age/underweight: sex, age at 18 month visit, calendar month of birth, trial arm, maternal height, maternal religion, household wealth. Weight-for-length/wasting: sex, age at 18 month visit, calendar month of birth, trial arm, maternal height, maternal parity, household wealth. MUAC-for-age Z-score: sex, age at 18 month visit, calendar month of birth, trial arm, maternal height, household wealth. Head circumference-for-age/microcephaly: sex, age at 18 month visit, calendar month of birth, trial arm, maternal age, maternal height, maternal religion, household wealth.

|  | **HIV positive** | | **HIV-exposed uninfected** | | | **HIV-unexposed** | |
| --- | --- | --- | --- | --- | --- | --- | --- |
|  | **Mean (SD) Z-score at 18 month visit** | | | | | | |
|  | **N** | **Z-score** | **N** | **Z-score** | **N** | | **Z-score** |
| **Length-for-age Z-score** | 11 | -3.13 (1.7) | 297 | -1.97 (1.1) | | 1771 | -1.58 (1.1) |
| **Weight-for-age Z-score** | 11 | -1.99 (1.1) | 297 | -0.93 (1.1) | | 1765 | -0.75 (1.0) |
| **Weight-for-length Z-score** | 11 | -0.50 (0.7) | 295 | -0.03 (1.1) | | 1762 | 0.02 (1.0) |
| **Mid-upper arm circumference-**  **for-age Z-score** | 11 | -0.67 (0.8) | 297 | -0.17 (0.9) | | 1758 | 0.01 (0.9) |
| **Head circumference-for-age**  **Z-score** | 11 | -0.74 (1.4) | 296 | -0.53 (1.1) | | 1757 | -0.26 (1.1) |
|  | **Dichotomous outcomes at 18 month visit** | | | | | | |
|  | **n/N** | **Percentage** | **n/N** | **Percentage** | **n/N** | | **Percentage** |
| **Stunting** | 7/11 | 64% | 150/297 | 51% | | 610/1771 | 34% |
| **Severe stunting** | 5/11 | 45% | 42/297 | 14% | | 155/1771 | 9% |
| **Underweight** | 5/11 | 45% | 48/297 | 16% | | 186/1765 | 11% |
| **Wasting** | 0/11 | 0% | 12/295 | 4% | | 47/1762 | 3% |
| **Microcephaly** | 2/11 | 18% | 29/296 | 10% | | 98/1757 | 6% |

**Supplementary Table 4. Growth outcomes of children who were HIV-positive, HIV-exposed uninfected, and HIV-unexposed children at 18 months of age.**

Stunting: length-for-age Z-score < -2; severe stunting: length-for-age Z-score < -3; underweight: weight-for-age Z-score < -2; wasting: weight-for-length Z-score < -2; microcephaly: head circumference-for-age Z-score < -2; 95%CI: 95% confidence interval. Data were missing if not measured or implausible values.

|  | **1mo**  **N=176 CHEU**  **N=1014 HIV-unexposed** | **3mo**  **N=166 CHEU**  **N=1061 HIV-unexposed** | **6mo**  **N=215 CHEU**  **N=1231 HIV-unexposed** | **12mo**  **N=220 CHEU**  **N=1318 HIV-unexposed** | **18mo**  **N=297 CHEU**  **N=1771 HIV-unexposed** |
| --- | --- | --- | --- | --- | --- |
| **LAZ; mean (SD)** |  |  |  |  |  |
| CHEU | -1.06 (1.11) | -1.08 (1.28) | -1.21 (1.27) | -1.61 (1.11) | -1.97 (1.07) |
| HIV-unexposed | -0.85 (1.23) | -0.86 (1.24) | -0.93 (1.18) | -1.27 (0.99) | -1.58 (1.07) |
| Difference (95%CI) | -0.21 (-0.38, -0.03) | -0.22 (-0.42, -0.03) | -0.27 (-0.47, -0.06) | -0.33 (-0.47, -0.19) | -0.38 (-0.51, -0.24) |
| **Stunting** |  |  |  |  |  |
| CHEU  Percentage (95%CI) | 31/176  18 (13, 24) | 26/166  16 (11, 22) | 60/215  28 (22, 34) | 72/220  33 (27, 39) | 150/297  51 (45, 56) |
| HIV-unexposed  Percentage (95%CI) | 166/1014  16 (14, 19) | 171/1061  16 (14, 18) | 216/1015  18 (16, 20) | 317/1318  24 (22, 26) | 610/1771  34 (32, 37) |
| Absolute difference, % | 1 (-5, 7) | 0 (-6, 6) | 10 (4, 17) | 9 (3, 15) | 16 (10, 22) |
| Relative risk | 1.09 (0.79, 1.50) | 0.97 (0.65, 1.43) | 1.57 (1.20, 2.07) | 1.36 (1.07, 1.74) | 1.46 (1.28, 1.67) |

|  | **1mo**  **N=175 CHEU**  **N=991 HIV-unexposed** | **3mo**  **N=160 CHEU**  **N=1049 HIV-unexposed** | **6mo**  **N=217 CHEU**  **N=1216 HIV-unexposed** | **12mo**  **N=221 CHEU**  **N=1308 HIV-unexposed** | **18mo**  **N=297 CHEU**  **N=1765 HIV-unexposed** |
| --- | --- | --- | --- | --- | --- |
| **WAZ; mean (SD)** |  |  |  |  |  |
| CHEU | -0.66 (1.08) | -0.68 (1.27) | -0.67 (1.21) | -0.89 (1.10) | -0.93 (1.01) |
| HIV-unexposed | -0.45 (1.91) | -0.39 (1.13) | -0.45 (1.14) | -0.62 (1.11) | -0.75 (1.12) |
| Difference (95%CI) | -0.20 (-0.39, -0.02) | -0.29 (-0.51, -0.07) | -0.22 (-0.40, -0.03) | -0.27 (-0.43, -0.12) | -0.17 (-0.32, -0.03) |
| **Underweight** |  |  |  |  |  |
| CHEU  Percentage (95%CI) | 19/175  11 (7, 16) | 23/160  14 (10, 21) | 32/217  15 (11, 20) | 39/221  18 (13, 23) | 48/297  16 (12, 21) |
| HIV-unexposed  Percentage (95%CI)  Absolute difference, % | 84/991  8 (7, 10)  2 (-3, 7) | 69/1049  7 (5, 8)  8 (2, 13) | 100/1216  8 (7, 10)  7 (2, 11) | 124/1308  9 (8, 11)  8 (3, 13) | 186/1765  11 (9, 12)  6 (1, 10) |
| Relative risk | 1.28 (0.79, 2.07) | 2.16 (1.37, 3.41) | 1.76 (1.18, 2.63) | 1.88 (1.36, 2.60) | 1.51 (1.12, 2.04) |

|  | **1mo**  **N=175 CHEU**  **N=984 HIV-unexposed** | **3mo**  **N=159 CHEU**  **N=1044 HIV-unexposed** | **6mo**  **N=215 CHEU**  **N=1212 HIV-unexposed** | **12mo**  **N=220 CHEU**  **N=1304 HIV-unexposed** | **18mo**  **N=295 CHEU**  **N=1762 HIV-unexposed** |
| --- | --- | --- | --- | --- | --- |
| **WLZ; mean (SD)** |  |  |  |  |  |
| CHEU | 0.41 (1.31) | 0.20 (1.49) | 0.18 (1.31) | -0.15 (1.15) | -0.03 (1.10) |
| HIV-unexposed | 0.43 (1.59) | 0.40 (1.36) | 0.22 (1.30) | -0.01 (1.22) | 0.02 (1.05) |
| Difference (95%CI) | -0.02 (-0.24, 0.20) | -0.21 (-0.45, 0.02) | -0.03 (-0.21, 0.16) | -0.14 (-0.30, 0.01) | -0.04 (-0.19, 0.11) |
| **Wasting** |  |  |  |  |  |
| CHEU  Percentage (95%CI) | 6/175  3 (2, 7) | 8/159  5 (3, 10) | 10/215  5 (3, 8) | 11/220  5 (3, 9) | 12/295  4 (2, 7) |
| HIV-unexposed  Percentage (95%CI)  Absolute difference, % | 61/984  6 (5, 8)  -3 (-6, 0) | 41/1044  4 (3, 5)  1 (-2, 5) | 50/1212  4 (3, 5)  1 (-3, 4) | 63/1304  5 (4, 6)  0 (-3, 3) | 47/1762  3 (2, 4)  1 (-1, 4) |
| Relative risk | 0.55 (0.22, 1.39) | 1.29 (0.63, 2.63) | 1.13 (0.56, 2.31) | 1.01 (0.58, 1.74) | 1.52 (0.78, 2.97) |

|  | **1mo** | **3mo**  **N=169 CHEU**  **N=1038 HIV-unexposed** | **6mo**  **N=214 CHEU**  **N=1232 HIV-unexposed** | **12mo**  **N=221 CHEU**  **N=1321 HIV-unexposed** | **18mo**  **N=296 CHEU**  **N=1757 HIV-unexposed** |
| --- | --- | --- | --- | --- | --- |
| **HCZ; mean (SD)** |  |  |  |  |  |
| CHEU |  | -0.32 (1.36) | -0.38 (1.28) | -0.48 (1.10) | -0.53 (1.12) |
| HIV-unexposed | N/A | -0.03 (1.26) | -0.12 (1.20) | -0.27 (1.12) | -0.26 (1.09) |
| Difference (95%CI) |  | -0.29 (-0.52, -0.05) | -0.27 (-0.43, -0.10) | -0.21 (-0.36, -0.06) | -0.27 (-0.40, -0.14) |
| **Microcephaly** |  |  |  |  |  |
| CHEU  Percentage (95%CI) |  | 17/169  10 (6, 16) | 18/214  8 (5, 13) | 22/221  10 (7, 15) | 29/296  10 (7, 14) |
| HIV-unexposed  Percentage (95%CI)  Absolute difference, % | N/A | 63/1038  6 (5, 8)  4 (-1, 9) | 77/1232  6 (5, 8)  2 (-2, 6) | 80/1321  6 (5, 8)  4 (0, 8) | 98/1757  6 (5, 7)  4 (1, 8) |
| Relative risk |  | 1.63 (0.99, 2.70) | 1.35 (0.86, 2.12) | 1.68 (1.09, 2.57) | 1.74 (1.18, 2.57) |

**Supplementary Table 5. Longitudinal growth outcomes of children who were HIV-exposed uninfected (CHEU) and children who were HIV-unexposed (CHU).**

CHEU: children HIV-exposed uninfected; CHU: children HIV-unexposed; LAZ: length-for-age Z-acore; WAZ: weight-for-age Z-score; WLZ: weight-for-length Z-score; HCZ: head circumference-for-age Z-score; stunting: length-for-age Z-score < -2; underweight: weight-for-age Z-score < -2; wasting: weight-for-length Z-score < -2; microcephaly: head circumference-for-age Z-score < -2; 95%CI: 95% confidence interval. Data were missing if not measured or implausible values.
